# Skyrmion motion driven by oscillating magnetic field

**DOI:** 10.1038/srep20360

**Published:** 2016-02-05

**Authors:** Kyoung-Woong Moon, Duck-Ho Kim, Soong-Geun Je, Byong Sun Chun, Wondong Kim, Z.Q. Qiu, Sug-Bong Choe, Chanyong Hwang

**Affiliations:** 1 Center for Nanometrology, Korea Research Institute of Standards and Science, Daejeon 305-340, Republic of Korea; 2 CSO and Department of Physics, Seoul National University, Seoul 151-742, Republic of Korea; 3 Department of Physics, University of California at Berkeley, Berkeley, California 94720, USA

## Abstract

The one-dimensional magnetic skyrmion motion induced by an electric current has attracted much interest because of its application potential in next-generation magnetic memory devices. Recently, the unidirectional motion of large (20 μm in diameter) magnetic bubbles with two-dimensional skyrmion topology, driven by an oscillating magnetic field, has also been demonstrated. For application in high-density memory devices, it is preferable to reduce the size of skyrmion. Here we show by numerical simulation that a skyrmion of a few tens of nanometres can also be driven by high-frequency field oscillations, but with a different direction of motion from the in-plane component of the tilted oscillating field. We found that a high-frequency field for small skyrmions can excite skyrmion resonant modes and that a combination of different modes results in a final skyrmion motion with a helical trajectory. Because this helical motion depends on the frequency of the field, we can control both the speed and the direction of the skyrmion motion, which is a distinguishable characteristic compared with other methods.

A magnetic skyrmion is a topological spin texture of which the spins wrap around a unit sphere^1^,^2^,^3^,^4^. A well-known example of skyrmion system is the chiral magnets in skyrmion crystal materials^4^,^5^. Recently, skyrmion control has been drawing much attention because of its use in noble spin devices as well as an unexpected underlying physics^6^,^7^,^8^,^9^,^10^,^11^,^12^,^13^. A precise control of the skyrmion motion relies on a full understanding of the skyrmion dynamics. The unidirectional motion of skyrmions, which can be realized by a magnetic field gradient^14^, temperature gradient^11^,^12^,^13^, and electric current flow in the sample^6^,^7^,^8^,^9^,^10^, is expected to be useful for data transfer in memory and logic devices.

Recently, it has been demonstrated that a tilted alternating magnetic field can also induce such a unidirectional motion^15^,^16^. The direction of this unidirectional motion was towards the tilted field angle. Note that in this report, relatively large circular bubble domains (~10 μm in size) were used rather than small skyrmions (~10 nm in size) and the applied magnetic field had low frequency (<1 kHz). In this paper, we expand our scope toward the small skyrmion beyond the large bubble and we expand the field frequency range up to GHz by employing micromagnetic simulations^17^,^18^. The field oscillation in the GHz frequency range can cause several resonant behaviours^19^,^20^,^21^,^22^,^23^,^24^ of the skyrmion, such as a breathing mode^19^,^21^ and a gyration mode^14^,^22^,^23^. We show that a combination of different resonance modes could not only drive the skyrmion motion but also control the skyrmion moving direction and speed by the field frequency and phase.

## Results

### Micromagnetic simulations

We performed micromagnetic simulations using the OOMMF code^17^ with the DMI extension module^18^. The material parameters were chosen as follows. The saturation magnetization *M*_*s*_ was 580 kA m^−1^, the exchange stiffness 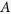 was 15 pJ m^−1^, the uniaxial magnetic anisotropy 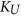 was 0.8 MJ m^−3^, and the Dzyaloshinskii–Moriya interaction^25^,^26^ (DMI) constant 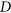 was −3.5 mJ m^−2^. Most of these parameters have been used in Ref. 8 for stable skyrmion texture. We used a relatively large damping constant 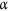 of 0.3 to keep the values of Pt/Co/Pt films^27^,^28^. However, the selection of large 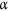 does not affect the main results (see Supplementary Fig. S1 for smaller 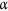 values). It is known if the skyrmion is confined to the magnetic disc, it exhibits flower-like trajectories^14^,^22^,^23^,^29^ and such bounded skyrmion does not show unidirectional motion. So, we assumed a magnetic disc structure with a relatively large radius (150 nm) compared with the skyrmion radius (~15 nm) to observe almost free skyrmion motions not bounded by the disc edge^8^,^14^,^22^,^23^,^29^. The disc thickness 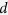 was set to 0.4 nm. The unit cell was selected as 2 nm × 2 nm × 0.4 nm.

### Skyrmion motion within an oscillatory magnetic field

**Figure 1:**
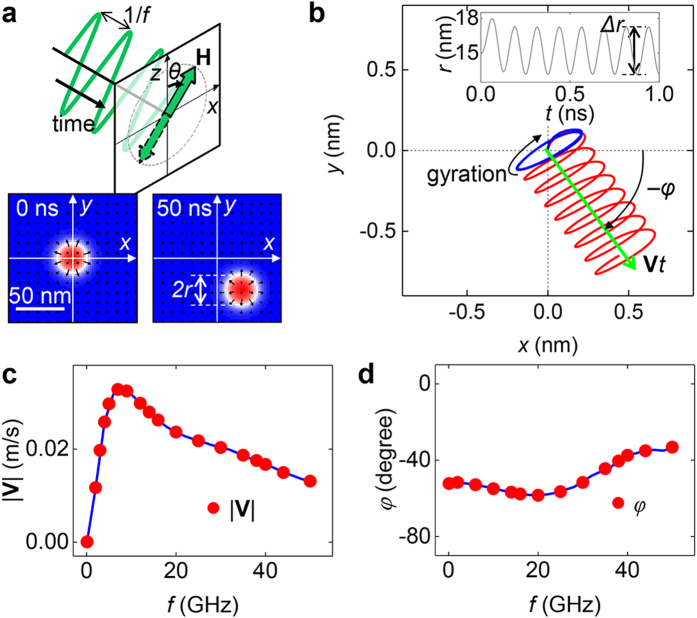
Skyrmion motion driven by uniaxial magnetic field oscillation. (**a**) The uniaxial field oscillation and the magnetization states of the centre of the disc. The field strength 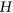 is 50 mT, the frequency 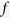 is 8 GHz, and the tilting angle 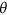 is 45°. The field is a sinusoidal function at time *t*. In magnetization images, the red region has perpendicular magnetization aligned along the +*z* direction and the blue region corresponds to the −*z* direction magnetization. The white colour denotes in-plane (*x–y* plane) magnetization and the black arrows represent in-plane magnetization directions. After application of the field during 50 ns, the skyrmion has shifted from its initial position. (**b**) The helical trajectory of the skyrmion centre (red line) from 0 ns to 1 ns obtained in (**a**). The blue line represents the gyration motion obtained via subtraction of the steady motion 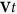 from the helical motion. The inset shows the skyrmion radius variation during the motion. **(c)** Speeds of the steady motion 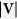 as a function of 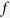 with 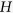 = 10 mT and 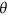 = 45°. **(d)** Directions of the skyrmion motion 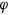 with respect to the field frequency.

The initial skyrmion state has its core at the centre of the disc with a +*z* directional polarity (up skyrmion). The spin texture in the central region of the disc is depicted in Fig. 1a. The red and blue colours represent magnetizations in the +*z* and −*z* directions, respectively. The white colour denotes the in-plane magnetization in the domain wall region with the black arrows indicating the in-plane magnetization direction. Because of the sufficient strength of DMI and the proper sign, the domain wall has radial outward magnetization^8^. The position of the skyrmion was determined by circular fitting of the domain wall positions. We defined the skyrmion radius 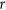 as the distance from the skyrmion centre to the domain wall position (see Methods).

An oscillating external magnetic field was applied to verify the skyrmion motion. The field 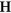 is spatially uniform with a sinusoidal time (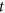) dependence, an amplitude of 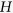 = 50 mT, and a frequency of 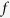 = 8 GHz. The field is titled in the *z*-*x* plane with the tilting angle 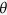 being 45° away from the *z*-axis. After the application of the field for 50 ns, the skyrmion was shifted ~40 nm from its initial position as shown in Fig. 1a. Thus, we confirmed that an oscillatory magnetic field induces a motion of the skyrmion. Now, we will present the detailed characteristics of the oscillating field-induced skyrmion motion.

Figure 1b shows the detailed trajectory (red line) of the skyrmion centre during the motion shown in Fig. 1a. The skyrmion exhibits a helical motion, from which we can extract the steady motion (green arrow) with the velocity 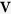 defined as the average motion of the skyrmion over a long time period. It is notable that 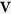 is not in the *x*-direction, which is different from the magnetic bubble motion^15^,^16^. We defined this skyrmion motion direction with the azimuthal angle 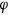 from the *x*-axis. Figure 1c,d show the skyrmion moving speed and direction as a function of the oscillatory field frequency at 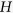 = 10 mT amplitude and 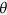 = 45°. The skyrmion moving reaches maximum speed near 8 GHz, indicating a resonance behaviour of the skyrmion under this condition. However, the moving direction does not exhibit any clear resonance behaviour but rather an almost monotonic dependence on 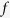.

Next, subtraction of the linear movement 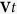 from the helical motion left the elliptic gyration with a clockwise rotation as shown by the blue line in Fig. 1b. It is well known that the sign of the topological charge *q* determines the rotation direction of the gyration and is typically determined by the polarity of the topological objects^2^,^14^,^22^,^23^. A prominent example is the gyrations of the vortex core. A magnetic vortex with +*z* (−*z*) polarity has a nonzero positive (negative) *q* thus should exhibit counter-clockwise (clockwise) gyration^30^,^31^,^32^. Similarly, the sign of a skyrmion charge *q* is determined by its polarity. According to previous studies^14^,^22^,^23^ a skyrmion with +*z* polarity should show counter-clockwise gyration, but our results show the opposite case, i.e. clockwise gyration.

To study the characteristics of the skyrmion motion mentioned above, we decomposed the tilted oscillating field into perpendicular (along the *z*-axis) and in-plane (along the *x*-axis) components to check the magnetization oscillation modes driven by each of these two components.

### Breathing motion of the skyrmion

**Figure 2:**
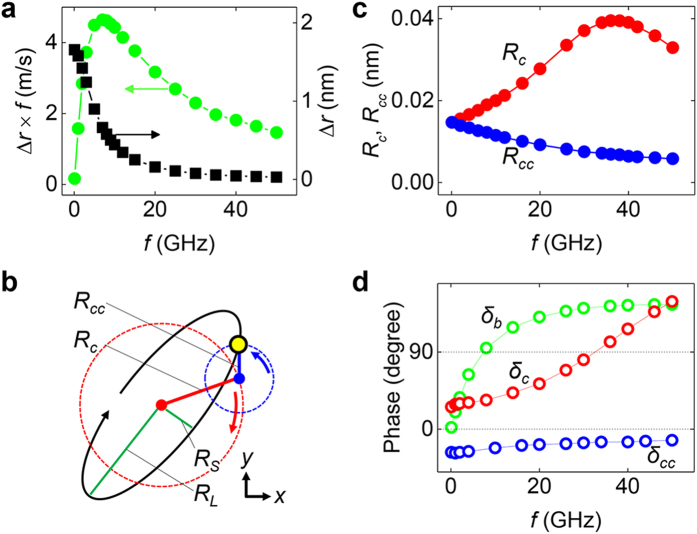
Resonant modes of the skyrmions. (**a**) Breathing amplitude (Δ*r*) and rates of the skyrmion radius variation (Δ*rf*) as a function of field frequency. To obtain this curve, the field was applied to the *z* direction with a strength of 5 mT. (**b**) Schematic diagram of the decomposition of the elliptic gyration into two circular gyrations having opposite rotating directions (clockwise, counter-clockwise). (**c**) The resonance curves for the two gyration modes. The field was applied to the 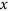 direction with a strength of 5 mT. (**d**) Phases of the breathing mode (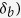 and the gyration modes (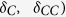.

The perpendicular field oscillation along the *z*-axis mainly causes the breathing motion of the skyrmion^19^,^21^. The radius variation during the skyrmion motion is shown in the inset of Fig. 1b and the radius variation has a saturated amplitude, Δ*r*, which represent the breathing mode amplitude. To obtain the breathing motion only, a sinusoidal field was applied in the *z* direction with an amplitude of *H* = 5 mT. Figure 2a depicts Δ*r* as well as the changing rates of radius in term of Δ*rf* which represents the amounts of radius change in unit time, i.e., the breathing speed. Among these two values (Δ*r* and Δ*rf*), it is notable that Δ*rf* shows more accordance with the speed of the skyrmion motion (Fig. 1c). Both the skyrmion speed and Δ*rf* have the maximum value near 8 GHz. This accordance implies that Δ*rf* should play an important role in determining the skyrmion moving speed.

### Gyration modes of the skyrmion

The in-plane field generates the gyration motion^19^,^22^,^23^,^29^,^32^,^33^. We applied an oscillating in-plane fields in the 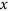-axis with an amplitude of 5 mT. After turning on this field, the skyrmion eventually reaches a steady gyration without an obvious drifting. As seen in Fig. 1b, the trajectory of the gyration is an ellipse rather than a circle. Thus, the skyrmion gyration can be decomposed into two circular gyrations of opposite rotations. Figure 2b depicts how the two circular gyrations of opposite rotations generate an elliptic gyration. Two radii of the major (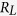) and minor (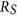) axes of the ellipse were measured, and then the radii of the two gyrations can be determined by 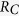 = (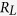
+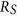)/2 and 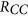 = (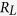
−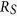)/2, where 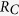 and 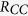 are the radius of the clockwise and counter-clockwise gyrations, respectively. Figure 2c exhibits the values of 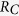 and 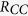 as a function of the field frequency. The clockwise gyration has a larger amplitude than the counter-clockwise gyration, resulting in a clockwise elliptic gyration of the skyrmion motion.

It was recently reported that a skyrmion confined in a circular disc has two kinds of gyration, clockwise and counter-clockwise^22^,^23^,^29^. The resonance frequencies of these two circular gyrations are determined by three parameters; the gyrocoupling vector strength 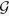, the spring constant 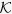, and the skyrmion mass 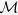. In our model, we assumed a non-bounded free skyrmion (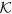 = 0). This condition results in two resonance angular frequencies, namely 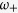 ~ 0 and 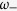 ~ 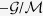, where 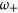 is for counter-clockwise gyration and 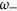 is for clockwise gyration with the up-polarity skyrmion. This situation is similar to that in Fig. 2c because 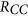 shows a monotonous decrease with increasing frequency because of the zero resonance frequency. However, 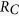 exhibits a resonance peak near 38 GHz, which corresponds to 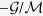. Such resonance behaviour is also shown in Fig. 2d, which shows the phases of the clockwise and counter-clockwise gyrations 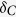 and 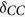, respectively. Note that 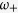 originates from the massless motion of the skyrmion^22^. We can name the circular counter-clockwise gyration “massless gyration.” In contrast, 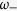 depends on the skyrmion mass so that we can name the circular clockwise gyration “massive gyration.”

### Skyrmion motion with circular gyrations

**Figure 3:**
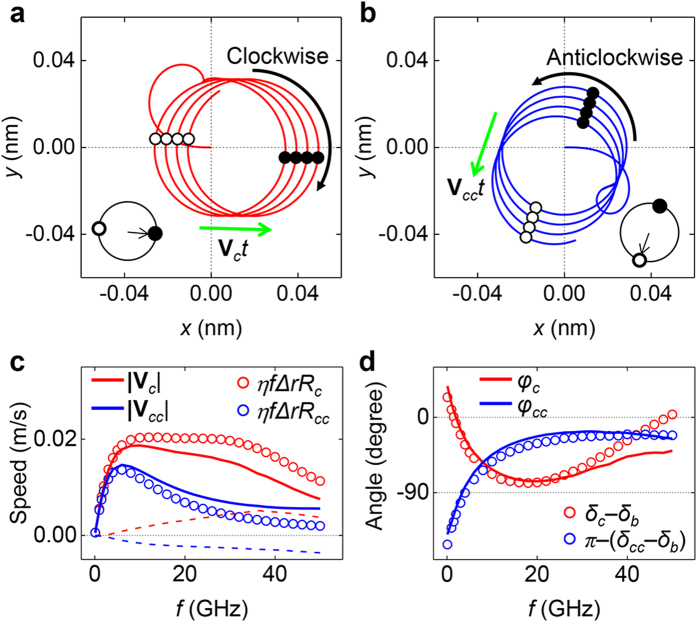
Skyrmion motion driven by rotating magnetic fields. (**a**) Trajectory of the skyrmion centre with clockwise gyration (red line). **(b)** Trajectory with counter-clockwise gyration (blue line). The magnetic field is (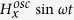, 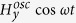, 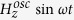), where 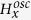 = 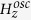 = 5 mT. 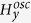 = 5 mT for (a) and 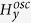 = −5 mT for **(b)**. 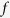 is 2 GHz. The black open (closed) circles represent the skyrmion centre positions where the skyrmion radius has the local maximum (minimum) value. **(c)** Speeds of skyrmion motion as a function of field frequency. The red (blue) dashed line denotes the speed difference between 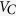 (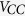) and 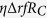 (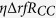). **(d)** Directions of skyrmion motion with respect to the field frequency and phase differences between the breathing and gyration modes.

To separate the massive and massless gyrations in the skyrmion motion, we applied a rotating in-plane field rather than a uniaxial in-plane field because a rotating field should induce only one of its gyrations. We define the external field as 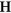 = (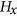, 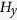, 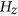) = (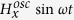, 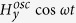, 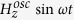), where 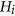 is the field component along the *i*-axis, 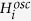 is the field amplitude in the *i* direction, and the angular frequency 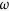 is 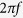. Figure 3a presents the skyrmion trajectory up to 2 ns when 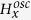 = 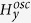 = 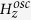 = 5 mT and 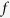 = 2 GHz. In this case, we observe clockwise circular gyration with a steady motion of constant velocity 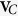. Changing the sign of 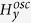, i.e. 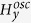 = −5 mT, the constant velocity 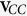 and the circular gyration are retained but the gyration switches to counter-clockwise because of the counter-clockwise field rotation, as shown in Fig. 3b.

These velocities depend strongly on the gyration radius as well as on 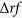. Figure 3c presents the absolute value of the velocities as a function of the field frequency. 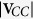 shows a simple resonance behaviour with a resonance peak near 8 GHz which is similar to 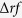 in Fig. 2a. However, 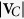 exhibits a plateau in the frequency range of 8–30 GHz; thus we expect that the gyration radius is also involved in determining 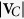. The 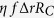 and 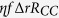 with a single fitting parameter, 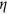 = 0.12 nm^−1^ (Fig. 3c) show similar behaviours as 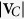 and 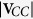. So, the multiples of each resonance amplitude and the field frequency roughly determine the skyrmion speed. However, there are notable discrepancies between 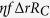 (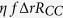) and 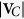 (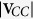) in the high-frequency regime. We think that the origin of this discrepancy (dashed lines in Fig. 3c) is related to the gyration radius because the discrepancy shows a similar trend to those of 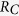 and 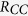 in Fig. 2c.

The moving direction of the skyrmion also shows a dependence on the field rotation direction. We plotted the moving direction as a function of the field frequency in Fig. 3d. The term 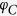 (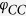) is the angle between the direction of 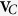 (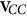) and the *x*-axis. We used the same definition of 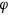 as in Fig. 1b. Note that the variation of 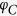 and 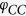 is wider than that of 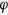. For example, the clockwise rotating field at 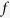 = 1.5 GHz induces a skyrmion motion along the +*x* direction, whereas the clockwise rotating field at 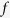 = 18 GHz induces an almost −*y*-directional motion. This result shows that the frequency of the rotating magnetic field can control the direction of the skyrmion movement within a relatively wide angular range, which is not possible in previous methods such as the electric current flow^6^,^7^,^8^,^9^,^10^ and the thermal or the field gradient^11^,^12^,^13^ methods.

The skyrmion moving direction depends strongly on the phase difference between the breathing mode and the circular gyration mode, as shown in Fig. 3d. This result indicates that the phase difference is the main factor in determining the skyrmion moving direction. The black closed (open) circles in Fig. 3a,b denote the positions where the skyrmion radius has its local maximum (minimum) values on the skyrmion trajectories. This result shows how the phase difference determines the skyrmion moving direction. In the clockwise (counter-clockwise) rotation case, the skyrmion shifts towards where it has its maximum (minimum) radius (insets of Fig. 3a,b).

## Discussion

The superposition of two skyrmion motions of clockwise and counter-clockwise gyrations gives rise exactly to a final skyrmion motion driven by an uniaxial oscillating field. In Fig. 1c it can be clearly seen that the 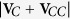 (blue line in Fig. 1c) exactly describes 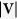. This result was also confirmed by comparing the moving direction of 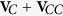 (blue line in Fig. 1d) with 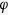.

**Figure 4:**
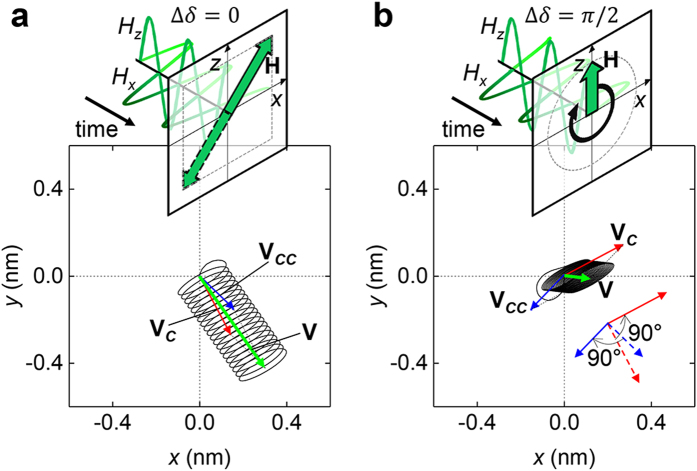
Skyrmion motions with different phase shifts between the breathing and the gyration modes. (**a)** Skyrmion trajectory induced by a linear field oscillation up to 2 ns. (**b**) Skyrmion trajectory induced by a field rotation up to 2 ns. The magnetic field is (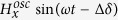, 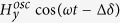, 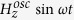), where 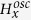 = 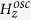 = 20 mT. 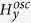 = 0 mT; 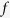 is 10 GHz; Δ*δ* was selected as 0 for (**a**) and π/2 for (**b**). The red and blue arrows denote 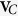 (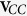) obtained by using 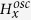 = (−)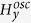 = 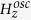 = 10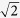 mT, and 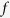 = 10 GHz. The dashed arrows in (**b**) represent 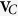 and 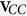 in (**a**).

Note that, it is possible to change the relative difference in the phase of the breathing and gyration modes by inserting a phase delay (Δ*δ*) in the field such as 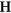 = (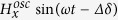, 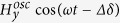, 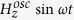). The term Δ*δ* directly affects the phase difference between breathing and gyration, and thus it should change the skyrmion motions. Figure 4a shows the helical trajectory and 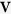 of the skyrmion motions driven by 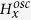 = 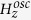 = 20 mT, 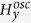 = 0, 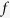 = 10 GHz, with Δ*δ* = 0. Such a linear oscillation of the field could be changed to a rotating field on the *x*-*z* plane when Δ*δ* = π/2, and this field results in a different skyrmion velocity (Fig. 4b). We can easily explain these two motions regardless of distinct motions of 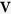. We obtained 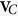 (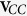) using 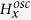 = (−)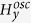 = 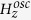 = 10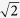 mT, and 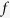 = 10 GHz and this is displayed in figures. The sum of 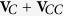 well explains 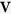 of both cases of Δ*δ* (= 0, π/2). The change in Δ*δ* from 0 to π/2 results in the rotation of the moving directions of 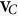 and 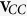 by π/2. However, the rotating directions of 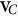 and 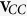 are opposites because two gyrations have opposite rotating directions.

**Figure 5:**
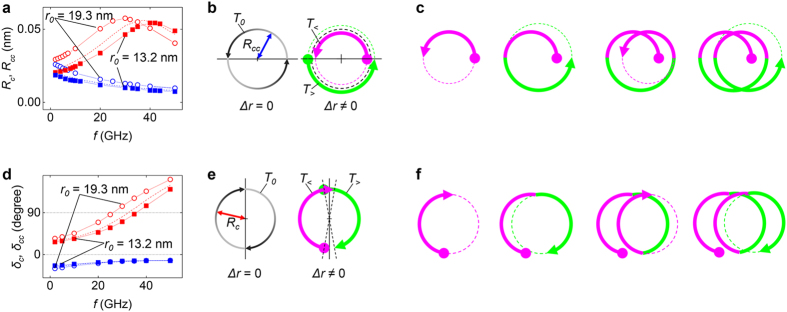
Two simple models for skyrmion motions. (**a**) Gyration amplitudes during the skyrmion motion with different skyrmion radii (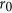) obtained by application of an additional external bias field, 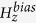. A common oscillating field (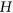 = 10 mT, 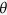 = 45°) was applied for the skyrmion motion. The open circles denote 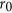 = 19.3 nm cases with 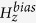 = 15 mT; the closed squares represent 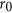 = 13.2 nm and 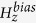 = −15 mT. The dashed lines are results obtained for 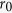 = 15 nm with 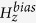 = 0; 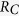 (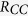) is represented as the red (blue) colour. (**b**) Schematic for counter-clockwise gyration during one period of radius oscillation with and without skyrmion radius variation. The black straight line denotes the positions where the switching of the skyrmion radii occurs. (**c**) Construction of the skyrmion trajectory by using two half-gyrations. (**d**) Phase variation of the gyration modes caused by the skyrmion radius; 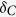 (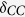) is represented as the red (blue) colour. Other indexing and simulation conditions are the same as those in (**a**). (**e**) A schematic of clockwise gyration during one period of the radius oscillation with and without the skyrmion radius variation. (**f**) Formation of the helical trajectory by using two half-gyrations.

The exact description of the skyrmion motion induced by field oscillation requires intricate analytic calculations. Instead, we show two simple descriptions of the origin of the helical trajectories as well as the determination of the skyrmion moving direction. These descriptions are based on the effect of the skyrmion radius on the gyration amplitudes. During the breathing motion, the skyrmion radius varied between the maximum and minimum values. To observe the effect of different radii on the gyration, we carried out simulations with an additional perpendicular bias field, 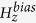, to change the mean value of the skyrmion radius^21^,^23^. Figure 5a shows the gyration amplitudes of skyrmions having different 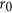 values (19.3, 15.0, and 13.2 nm) with a common oscillating field condition (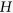 = 10 mT, 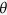 = 45°). Each 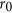 value was obtained with a different 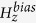 (15, 0, and −15 mT). The larger skyrmion has a lower resonance frequency and larger amplitude.

We supposed that this effect of the skyrmion radii on the gyration amplitudes also occurred during the breathing motion. To make the description simple, we assumed that the breathing skyrmion only has two states having two different radii, 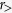 (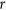
>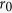) and 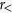 (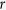
<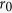), and discontinuous changes of states occur at 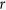 = 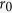. Each radius state should determine the gyration trajectories during each half gyration. Figure 5b presents an example in which the one cycle of the initial circular trajectory, 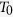, was divided into two half circular trajectories (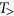 and 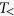) because of the variation in 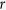. Here, 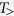 (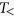) denotes the trajectory of the half gyration with 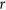 = 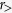 (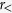). We could create a helical motion by successive arrangement of 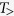 and 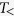 (Fig. 5c). The other similar description is also possible by using the phase variation of the gyration mode as shown in Fig. 5d. As the resonance frequency shifts because of a different 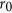
value, the phase also shifts. Figure 5e depicts the situation in which the radius oscillation changes the phase of each half period of clockwise gyration, and then, successive connections of these two half gyrations form the helical trajectory (Fig. 5f).

These two simple models roughly describe the speed determination as well as the moving direction of the skyrmion. The skyrmion positions on the gyration trajectory when 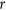 = 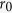 are determined by the phase difference between the breathing and the gyration modes, and that position is directly related to the skyrmion moving direction (Fig. 5b,c,e,f). In addition, it is obvious that the speed of skyrmion is proportional to the amplitude of gyration. The speed should also have linear dependence on *f*Δ*r* in the linear regime of the phases shifts and on the gyration amplitudes changes with Δ*r*. However, these models have a number of weak points. The clockwise gyration amplitude stops varying linearly near the resonance frequency (Fig. 5a). Moreover, the expected moving directions differ from those of Fig. 3 by a constant angle, *π*/2 or *π*. Therefore, a more accurate model is required.

**Figure 6:**
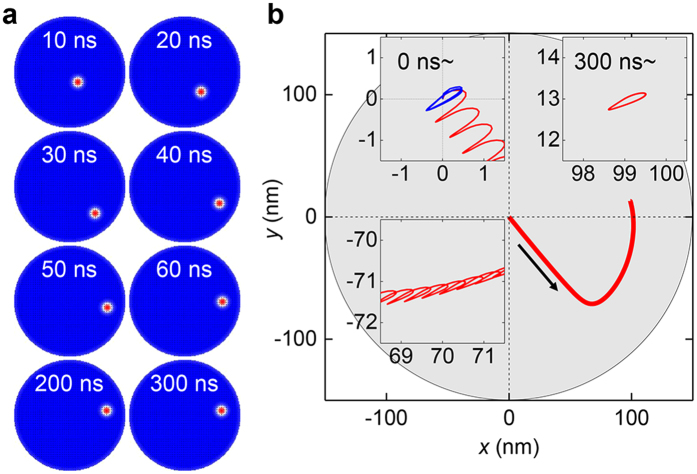
Skyrmion motions for the longer period with H = 0.1 T and f = 7 GHz. (**a**)Magnetization states on the entire disc during the motions. Steady motions are observed up to 20 ns. When the skyrmion approaches the disc boundary (30 ns), the skyrmion does not escape the disc and changed its moving direction along the disc edge. During this motion (30 ~200 ns), the skyrmion speed gradually reduce to 0. (**b**)Skyrmion trajectories (red line) on the magnetic disc. The gray circle indicates the disc area. Insets show detailed trajectories (initial motion, changing the moving direction, and after stop).

To study an interaction between the skyrmion and the disc edge, we studied the skyrmion motion on longer time scales. It was known that the edge of the magnetic material exerts a repulsive force that confines the skyrmion in the material^8^,^14^,^22^,^23^,^29^. Figure 6 represents the skyrmion trajectory in the entire disc under the field condition (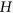 = 100 mT, 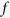 = 7 GHz, and 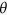 = 45°). The initial motion with a linear velocity maintained for 30 ns and then the skyrmion shows tracking motion along the disc edge. During the tracking motion, the skyrmion speed gradually reduces and finally goes to zero. The stopped skyrmion after 300 ns exhibits the gyration motion around a new equilibrium position (inset of Fig. 6b). Interesting thing is the gyration of the stopped skyrmion is not significantly different form the free skyrmion gyration (blue line in the inset of Fig. 6b) and the Δ*r* also has similar value (9.5 nm for the free skyrmion and 9.4 nm for the stopped skyrmion). Thus, we think that the disc edge directly exerts a repulsive force to the skyrmion rather than changes the skyrmion oscillation amplitudes.

To summarize, we studied skyrmion motion within an oscillating magnetic field by micromagnetic simulations. A tilted uniaxial oscillating field produced the breathing and gyration modes, and the combination of these two modes resulted in a skyrmion motion with a helical trajectory. This motion could be decomposed into a clockwise and a counter-clockwise gyration, which are related to the massive and massless gyration modes, respectively. We studied these two skyrmion motions and found the resonance behaviours that determine the skyrmion speed and moving direction. The skyrmion motion induced by field oscillation has controllability of the direction of movement with frequency, which is a characteristic that is distinguishable from other methods.

## Methods

### Skyrmion positions, radii, and velocities

We obtained the skyrmion positions, radii, and velocities as follows. First, we extracted the interpolated values of the normalized perpendicular magnetization (*m*_z_) of the *i*-th magnetization state along a line from (*x*_*i*−1_, *y*_*i*−1_) to a given direction, *ψ*, on the *x*,*y* plane with an interval of 2 nm. Here, (*x*_*i*−1_, *y*_*i*−1_) is the centre position of the (*i*−1)-th state. Then, we fitted a hyperbolic tangent function in *m*_z_ to determine the domain wall position where *m*_z_ = 0. Finally, the circular fitting of every wall position obtained on every *ψ* produces the skyrmion centre (*x*_*i*_, *y*_*i*_) and the average of the distances form the centre to the wall positions results in the skyrmion radius (see Supplementary Fig. S2). To determine the velocity (speed and moving direction) of skyrmion motions, we extracted interpolated skyrmion positions with a fixed time interval (1/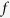), and linear fitting of these positions resulted in the skyrmion velocities (see Supplementary Fig. S3). The error bar in position was less than 0.01 pm. During skyrmion motions, there is no significant deformation of the skyrmion shape even with a large field, 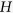 = 0.1 T (see Supplementary Fig. S4). Note that if we observe the skyrmion motion for a longer period, we can see a bounded skyrmion motion tracking the disc boundary (Fig. 6). Thus, the skyrmion motions were observed only near the centre of the disc.

### Selection of the field angle

In the simple linear regime, the *z*-directional field component directly determines the breathing mode amplitude, and similarly, the gyration amplitudes show linear dependences on the *x*-directional field component. Therefore, the amplitudes of the breathing and the gyration modes should have 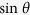 and 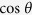 behaviours, respectively. Because a multiple of 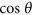 and 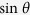 has a maximum at 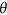 = 45°, we can expect that the maximum skyrmion speed also occurs at 45° and this expectation is well verified (see Supplementary Fig. S5).

## Additional Information

**How to cite this article**: Moon, K.-W. *et al.* Skyrmion motion driven by oscillating magnetic field. *Sci. Rep.* **6**, 20360; doi: 10.1038/srep20360 (2016).

## Supplementary Material

Supplementary Information

## References

[b1] Skyrme T. H. R. A unified field theory of mesons and baryons. Nucl. Phys. 31, 556–569 (1962).

[b2] Heinze S. *et al.* Spontaneous atomic-scale magnetic skyrmion lattice in two dimensions. Nat. Phys. 7, 713–718 (2011).

[b3] Pfleiderer C. Surfaces get hairy. *Nat. Phys*. 7, 673–674 (2011).

[b4] Nagaosa N. & Tokura Y. Topological properties and dynamics of magnetic skyrmions. Nat. Nanotech. 8, 899–911 (2013). 10.1038/nnano.2013.243 24302027

[b5] Mühlbauer S. *et al.* Skyrmion lattice in a chiral magnet. Science 323, 915–919 (2009). 19213914 10.1126/science.1166767

[b6] Jonietz F. *et al.* Spin transfer torques in MnSi at ultralow current densities. Science 330, 1648–1651 (2010). 21164010 10.1126/science.1195709

[b7] Tomasello R. *et al.* A strategy for the design of skyrmion racetrack memories. Sci. Rep. 4, 6784 (2014). 25351135 10.1038/srep06784 PMC4212245

[b8] Sampaio J., Cros V., Rohart S., Thiaville A. & Fert A. Nucleation, stability and current-induced motion of isolated magnetic skyrmions in nanostructures. Nat. Nanotech. 8, 839–844 (2013). 10.1038/nnano.2013.210 24162000

[b9] Nagaosa N. & Tokura Y. Topological properties and dynamics of magnetic skyrmions, Nat. Nanotech. 8, 899–911 (2013). 10.1038/nnano.2013.243 24302027

[b10] Iwasaki J., Mochizuki M. & Nagaosa N. Universal current-velocity relation of skyrmion motion in chiral magnets. Nat. Commun. 4, 1463 (2013). 23403564 10.1038/ncomms2442

[b11] Mochizuki M. *et al.* Thermally driven ratchet motion of a skyrmion microcrystal and topological magnon Hall effect. Nat. Mater. 13, 241–246 (2014). 24464244 10.1038/nmat3862

[b12] Kong L. & Zang J. Dynamics of an insulating skyrmion under a temperature gradient. Phys. Rev. Lett. 111, 067203 (2013). 23971607 10.1103/PhysRevLett.111.067203

[b13] Everschor K. *et al.* Rotating skyrmion lattices by spin torques and field or temperature gradients. Phys. Rev. B 86, 054432 (2012).

[b14] Moutafis C., Komineas S. & Bland J. A. C. Dynamics and switching processes for magnetic bubbles in nanoelements. Phys. Rev. B 79, 224429 (2009).

[b15] Moon K.-W. *et al.* Magnetic bubblecade memory based on chiral domain walls. Sci. Rep. 5, 9166 (2015). 25772606 10.1038/srep09166 PMC5390916

[b16] Petit D., Seem P. R., Tillette M., Mansell R. & Cowburn R. P. Two-dimensional control of field-driven magnetic bubble movement using Dzyaloshinskii–Moriya interactions. Appl. Phys. Lett. 106, 022402 (2015).

[b17] Donahue M. J. & Porter D. The Object Oriented MicroMagnetic Framework (OOMMF) project at ITL/NIST. Available at: http://math.nist.gov/oommf (Accessed: 8^th^ October 2014).

[b18] Rohart S. & Thiaville A. Skyrmion confinement in ultrathin film nanostructures in the presence of Dzyaloshinskii-Moriya interaction. Phys. Rev. B 88, 184422 (2013).

[b19] Mochizuki M. Spin-wave modes and their intense excitation effects in skyrmion crystals. Phys. Rev. Lett. 108, 017601 (2012). 22304290 10.1103/PhysRevLett.108.017601

[b20] Vukadinovic N. & Boust F. Three-dimensional micromagnetic simulations of multidomain bubble-state excitation spectrum in ferromagnetic cylindrical nanodots. Phys. Rev. B 78, 184411 (2008).

[b21] Kim J.-V. *et al.* Breathing modes of confined skyrmions in ultrathin magnetic dots. Phys. Rev. B 90, 064410 (2014).

[b22] Makhfudz I., Krüger B. & Tchernyshyov O. Inertia and chiral edge modes of a skyrmion magnetic bubble. Phys. Rev. Lett. 109, 217201 (2012). 23215611 10.1103/PhysRevLett.109.217201

[b23] Moon K.-W., Chun B. S., Kim W., Qiu Z. Q. & Hwang C. Control of skyrmion magnetic bubble gyration. Phys. Rev. B 89, 064413 (2014).

[b24] Lin S.-Z., Batista C. D. & Saxena A. Internal modes of a skyrmion in the ferromagnetic state of chiral magnets. Phys. Rev. B 89, 024415 (2014).

[b25] Dzyaloshinskii I. A thermodynamic theory of ‘weak’ ferromagnetism of antiferromagnetics. J. Phys. Chem. Solids 4, 241–255 (1958).

[b26] Moriya T. Anisotropic superexchange interaction and weak ferromagnetism. Phys. Rev. 120, 91–98 (1960).

[b27] Mizukami S. *et al.* Gilbert damping in perpendicularly magnetized Pt/Co/Pt films investigated by all-optical pump-probe technique. Appl. Phys. Lett. 96, 152502 (2010).

[b28] Metaxas P. J. *et al.* Creep and flow regimes of magnetic domain-wall motion in ultrathin Pt/Co/Pt films with perpendicular anisotropy. Phys. Rev. Lett. 99, 217208 (2007). 18233251 10.1103/PhysRevLett.99.217208

[b29] Dai Y., Wang H., Yang T., Ren W. & Zhang Z. Flower-like dynamics of coupled Skyrmions with dual resonant modes by a single-frequency microwave magnetic field. Sci. Rep. 4, 6153 (2014). 25141993 10.1038/srep06153 PMC4139943

[b30] Van Waeyenberge B. *et al.* Magnetic vortex core reversal by excitation with short bursts of an alternating field. Nature 444, 461–464 (2006). 17122851 10.1038/nature05240

[b31] Choe S.-B. *et al.* Vortex core-driven magnetization dynamics. Science 304, 420–422 (2004). 15087545 10.1126/science.1095068

[b32] Kim S.-K., Lee K.-S., Yu Y.-S. & Choi Y.-S. Reliable low-power control of ultrafast vortex-core switching with the selectivity in an array of vortex states by in-plane circular-rotational magnetic fields and spin-polarized currents. Appl. Phys. Lett. 92, 022509 (2008).

[b33] Thiele A. A. Steady-state motion of magnetic domains. Phys. Rev. Lett. 30, 230–233 (1973).

