# Supplementary material for: Skyrmion motion driven by oscillating magnetic field
Source: Sci Rep. 2016 Feb 5;6:20360. doi: 10.1038/srep20360 (PMC4742868; doi:10.1038/srep20360)
Supplement: Supplementary Information [file srep20360-s1.pdf]

Supplementary Information for

Skyrmion motion driven by oscillating magnetic field

Kyoung-Woong Moon, Duck-Ho Kim, Soong-Geun Je, Byong Sun Chun, Wondong Kim,

Z.Q. Qiu, Sug-Bong Choe & Chanyong Hwang

This PDF file includes:

Supplementary Figs. S1 to S5

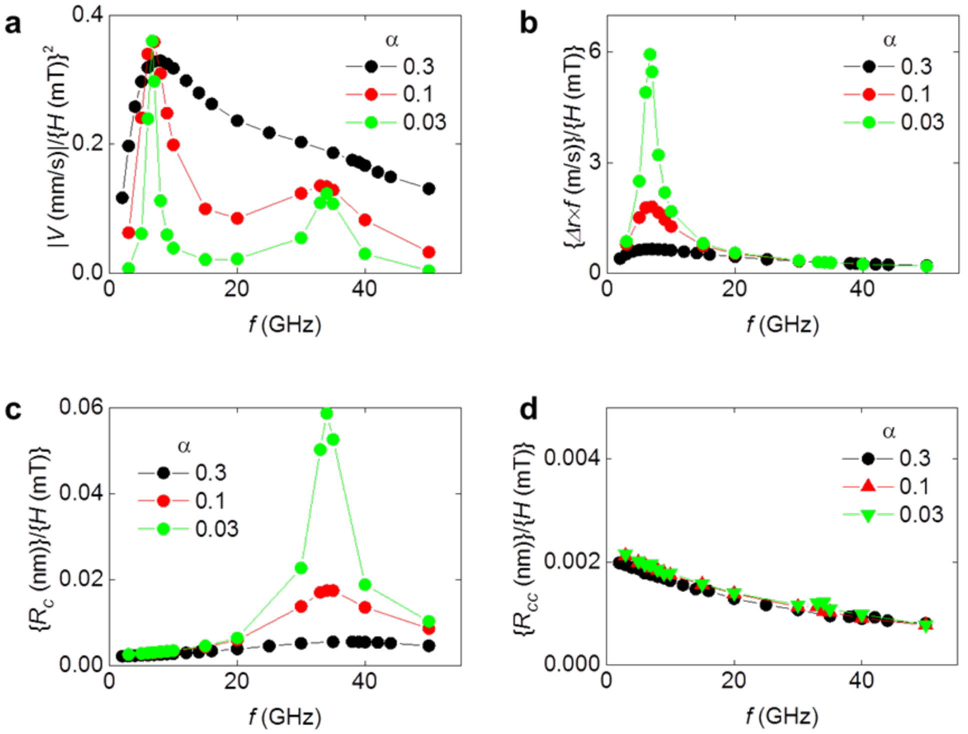

Supplementary Fig. S1 | Results with different *α*. To check the effect of various magnitudes of *α* while suppressing the nonlinear effect, *α* and the amplitude of oscillating field *H* were selected as follows; (*α* =0.3, *H* = 10 mT), (*α* =0.1, *H* = 3 mT), and (*α* =0.03, *H* = 1 mT). **(a)** Normalized skyrmion speeds by H^2 as a function of *f*. **(c)** Normalized breathing speed by *H*. **(c)** Normalized R_c by *H*. **(d)** Normalized R_{cc} by *H*.

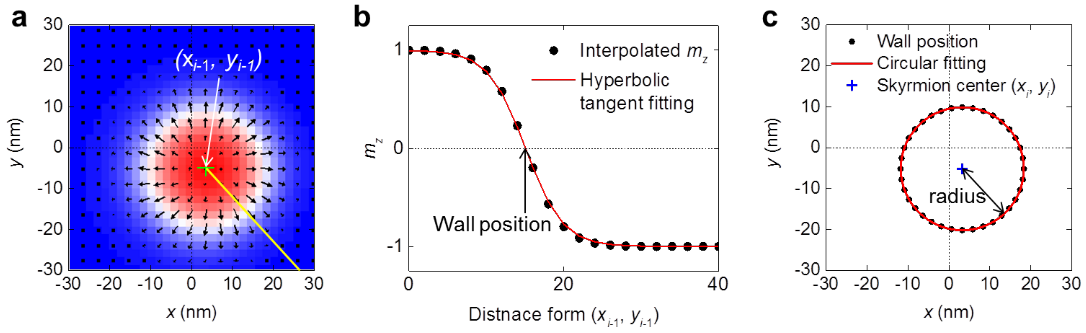

Supplementary Fig. S2 | A determination example of the skyrmion centre and its radius. **(a)** The i-th magnetization state and the skyrmion centre of the (i-1)-th magnetization state. **(b)** Interpolated values of m_z along the yellow line in (a) and a fitting result. **(c)** Wall positions, a circular fitting of wall positions, the skyrmion centre, and the radius of skyrmion.

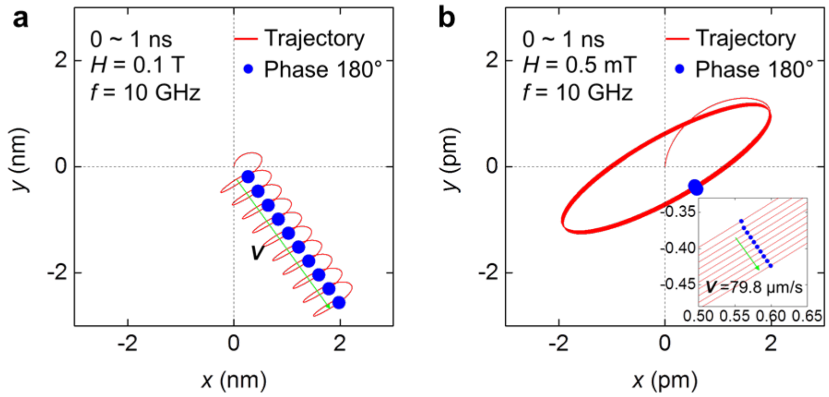

Supplementary Fig. S3 | Velocity determinations for skyrmion motions. The red lines are the skyrmion trajectory and blue points are skyrmion positions with fixed time interval ($1/f$ = 0.1 ns). **(a)** H = 0.1 T and f = 10 GHz. **(b)** H = 0.0005 T and f = 10 GHz.

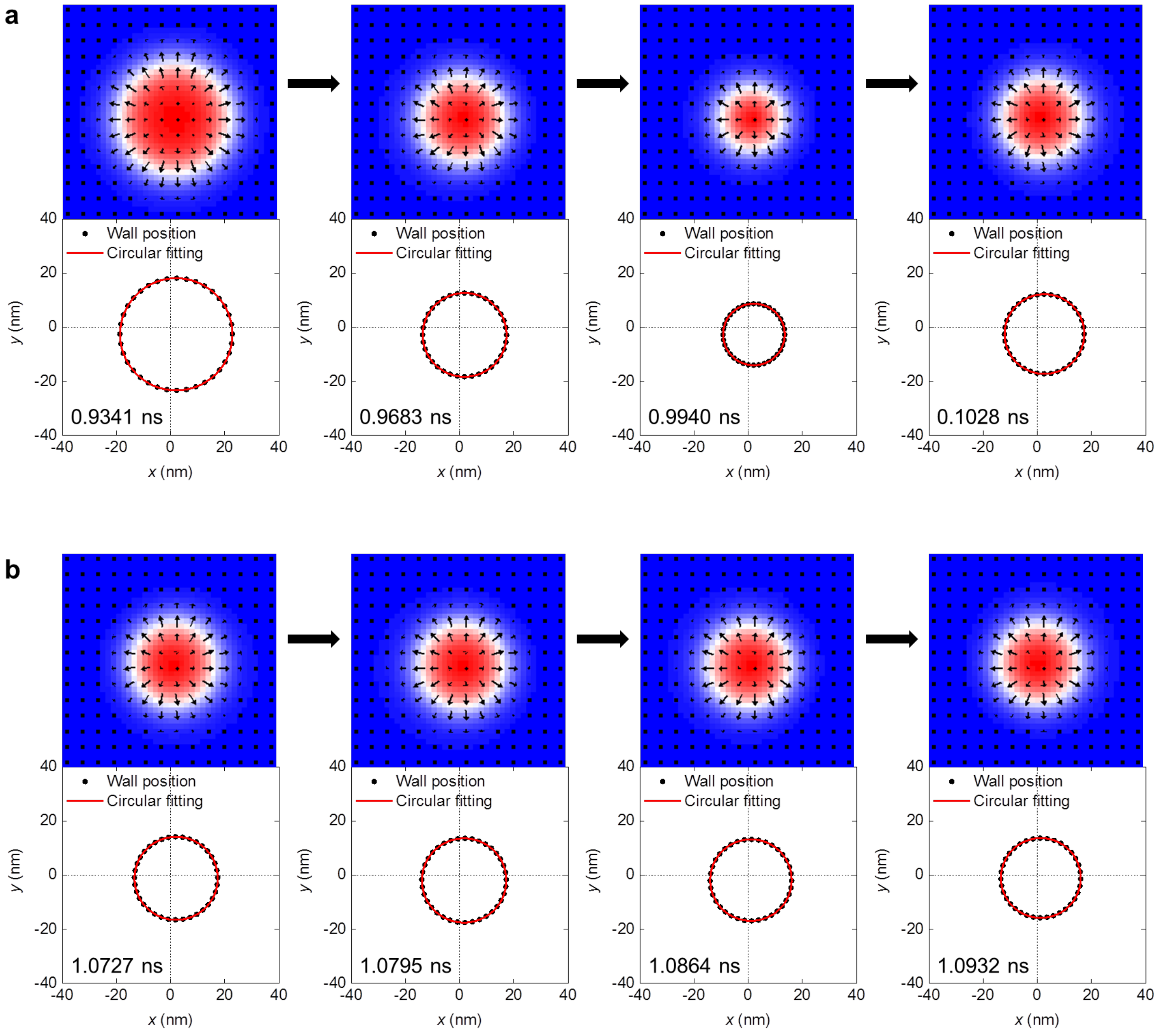

Supplementary Fig. S4 | Shape of skyrmions during the motion. (a) $H = 0.1$ T and $f = 7$ GHz. **(b)** $H = 0.1$ T and $f = 34$ GHz.

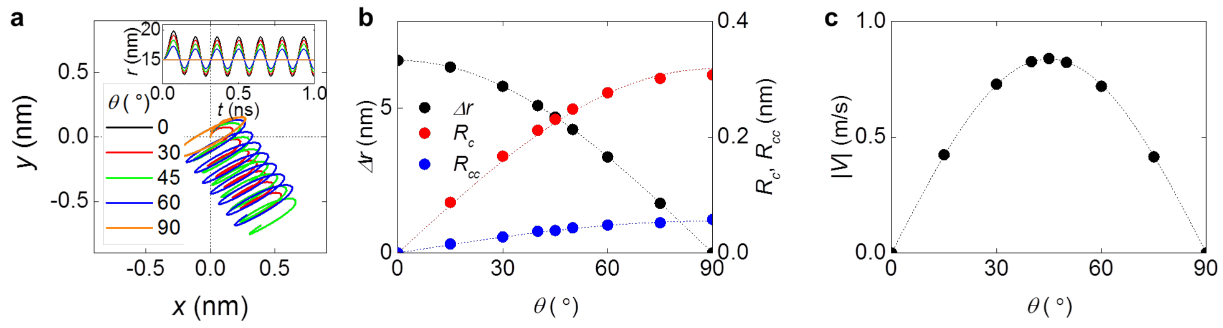

Supplementary Fig. S5 | Skyrmion motions as a function of field angle, θ with H = 0.05 T and f = 7 GHz. (a) Skyrmion trajectories and radius variations. **(b)** Amplitudes of the radius variation and radii of two gyrations. Dashed lines represent simple cosθ and sinθ fitting curves. **(c)** Skyrmion speeds as a function of θ. Dashed line is a simple cosθ·sinθ fitting result.
